# Supplementary material for: Content-rich biological network constructed by mining PubMed abstracts
Source: BMC Bioinformatics. 2004 Oct 8;5:147. doi: 10.1186/1471-2105-5-147 (PMC528731; doi:10.1186/1471-2105-5-147)
Supplement: Additional File 2 — The original results of the above study (non-essential files are deleted to keep the file size under the limit set by BMC bioinformatics). [file 1471-2105-5-147-S2.bz2 › chilibotAdditionalFile2/dip20/465ID9660793E10492/html/CXCR3_CXCL10.html]

 


 **CXCR3** and **CXCL10** 
  
Found 773 abstracts in PubMed, retrieved 20.  
 

 What does Google say? 
 PDF only 
| .edu only 

---

**Interactive relationship** (e.g. stimulation, inhibition, etc)

**Neutral relationship**- The chemokine receptor  **CXCR3**  is predominantly expressed on T lymphocytes, and its agonists CXCL9,  **CXCL10**  and CXCL11 are IFN gamma inducible chemokines that promote Th1 responses.  Ref: 12884299 Eur J Immunol, 2003
- The present findings suggest that MCP 1, RANTES, and IP 10  [ **CXCL10** ]   [ **CXCR3** ]  may participate in the pathogenesis of mercuric chloride induced tubulointerstitial fibrosis with mononuclear cell infiltration, via CCR2, CCR1 or CCR5, and  **CXCR3** , respectively.  Ref: 12834626 Exp Mol Pathol, 2003

**Non-interactive relationship** (e.g. studied together, co-existance, homology, etc.)

- AIM To assess the immunological role of human milk by analysing the concentrations of interferon gamma inducible protein of 10 kda IP 10  [ **CXCL10** ]   [ **CXCR3** ]  and monokine induced by interferon gamma MIG in human milk from mothers of preterm and term infants.  Ref: 12856973 Acta Paediatr, 2003
- Correspondingly, the rate or intensity of inflammation in IL 10 mice treated with anti IP 10  [ **CXCL10** ]   [ **CXCR3** ]  Abs showed improved scoring of inflammation, compared with control IL 10 mice.  Ref: 12874231 J Immunol, 2003
- IP 10  [ **CXCL10** ]   [ **CXCR3** ]  and MIG mRNA expression levels in cellular components of human milk were determined by RT PCR.  Ref: 12856973 Acta Paediatr, 2003
- This study provides important and novel information regarding IP 10  [ **CXCL10** ]   [ **CXCR3** ]  as a target for the treatment of colitis.  Ref: 12874231 J Immunol, 2003
- Potential molecular differences between early and late rejection grade > =2 episodes were examined by testing chemokine and receptor gene expression.METHODS Expression of inducible protein IP 10  [ **CXCL10** ]   [ **CXCR3** ] , monokine induced by IFN gamma Mig, interferon inducible T cell alpha chemoattractant I TAC, regulated on activation normal T cell expressed and secreted RANTES, and their receptors  **CXCR3**  and CCR5 was tested in 60 endomyocardial biopsies from 24 patients using quantitative Taqman real time polymerase chain reaction PCR.  Ref: 12829909 Transplantation, 2003
- Interferon gamma inducible protein 10  [ **CXCL10** ]  IP10  [ **CXCL10** ]   [ **CXCR3** ]  and monokine induced by interferon gamma MIG positive H& ; RS cells were detected in all 10 HL MC but only in 5 of 10 cases of HL NS and 2 of 9 cases of HL like ATLL.  Ref: 12866030 Int J CancerInt J Cancer, 2003
- In addition, IL 12 inhibits tumor angiogenesis mainly through IFNgamma dependent production of the chemokine IP10  [ **CXCL10** ]   [ **CXCR3** ] .  Ref: 12871184 Curr Pharm Des, 2003
- CCR3 functional responses are regulated by both  **CXCR3**  and its ligands CXCL9,  **CXCL10**  and CXCL11.  Ref: 12884299 Eur J Immunol, 2003
- Consistent with these results, infected cells from WT mice preferentially migrated in response to IP 10  [ **CXCL10** ]   [ **CXCR3** ]   **CXCR3**  ligand, while those from GKO mice migrated in response to eotaxin CCL11 CCR3 ligand.  Ref: 12875978 Am J Pathol, 2003
- DESIGN AND PATIENTS Immunocytochemistry and reverse transcriptase polymerase chain reaction RT PCR amplification were used to analyse the expression of monocyte chemoattractant protein MCP 1, RANTES regulated upon activation, normal T cell expressed and secreted, macrophage inflammatory protein MIP 1alpha, MIP 1beta, interferon IFN gamma inducible protein IP 10  [ **CXCL10** ]   [ **CXCR3** ]  and monokine induced by IFN gamma Mig in thyroid tissue from patients with Hashimoto s thyroiditis n = 4, Graves disease n = 6 and nonautoimmune multinodular goitre n = 4.  Ref: 12864798 Clin Endocrinol (Oxf), 2003
- CONCLUSION We postulate that TFC may play a role in the pathogenesis of autoimmune thyroid disease as they are able to express the chemokines MIP 1alpha, MIP 1beta, MCP 1, RANTES, IP 10  [ **CXCL10** ]   [ **CXCR3** ]  and Mig that would promote the infiltration of immune cells into the thyroid gland.  Ref: 12864798 Clin Endocrinol (Oxf), 2003
- Infiltrating CD8 T Cells in Oral Lichen Planus Predominantly Express CCR5 and  **CXCR3**  and Carry Respective Chemokine Ligands RANTES CCL5 and IP 10  [ **CXCL10** ]   [ **CXCR3** ]   **CXCL10**  in Their Cytolytic Granules A Potential Self Recruiting Mechanism.  Ref: 12819030 Am J Pathol, 2003
- We investigated the potential down regulators of chemokine secretion IL 4, IL 10, IL 13, dexamethasone, and PGE2, IL 10 and IL 13 down regulate M. tuberculosis induced IL 8, IP 10  [ **CXCL10** ]   [ **CXCR3** ] , RANTES, and MCP 1 secretion from MG 63 cells by between 48 and 94% P <.  Ref: 12797540 J Clin Immunol, 2003
- Furthermore, infiltrating T cells, particularly CD8 T cells, were positive for RANTES CCL5 and IP 10  [ **CXCL10** ]   [ **CXCR3** ]   **CXCL10** , the ligands of CCR5 and  **CXCR3** , respectively.  Ref: 12819030 Am J Pathol, 2003
- Specifically, IP 10  [ **CXCL10** ]   [ **CXCR3** ]  inhibition in IL 10 mice attenuates the associated increases in serum and or local amyloid A, IL 2, IL 6, TNF alpha, IFN gamma, IL 1alpha, and IL 1beta with colitis as compared with IL 10 mice that develop colitis similar to human Crohn s disease.  Ref: 12874231 J Immunol, 2003
- This work compares the proliferative, IgM, IL 6 and IP 10  [ **CXCL10** ]   [ **CXCR3** ]  response of PBMC from normal donors to a diverse panel of CpG ODNs.  Ref: 12869028 Clin Exp Immunol, 2003
- Biopsies were stained for CD68, CD3, elastase, eotaxin, IP 10  [ **CXCL10** ]   [ **CXCR3** ] , MCP 1, MCP 3, and IL 8 and analyzed by NIH Image analyzer.  Ref: 12827410 Int J Colorectal Dis< MedlineTA>Int J Colorectal Dis, 2003
- Kupffer cell blockade significantly reduced liver TNF alpha, MIP 2, and IP 10  [ **CXCL10** ]   [ **CXCR3** ]  gene expression and liver inflammation after the administration of AdL.PB\* or AdL.F\*PB\*.  Ref: 12804145 Hum Gene Ther, 2003
- Expression of IP 10  [ **CXCL10** ]   [ **CXCR3** ]  and MIG genes and proteins in the milk cells as well as in mammary gland epithelial tissues was detected by RT PCR and immunohistochemistry.  Ref: 12856973 Acta Paediatr, 2003
- The binding to IP 10  [ **CXCL10** ]   [ **CXCR3** ]  could be inhibited by SLC but not by a non mac25 AGM binding chemokine, EBI1 ligand chemokine ELC.  Ref: 12847218 J Immunol, 2003
- The binding of mac25 AGM to SLC and IP 10  [ **CXCL10** ]   [ **CXCR3** ]  was dose dependent and saturable.  Ref: 12847218 J Immunol, 2003
- Our findings show that IP 10  [ **CXCL10** ]   [ **CXCR3** ]  significantly contributes to the development of Th1 and inflammatory responses.  Ref: 12874231 J Immunol, 2003
- 2 Interferon IFN inducible anti viral proteins, including IFIT1, OAS1 and Mx1, and Th1 chemoattractant IP 10  [ **CXCL10** ]   [ **CXCR3** ] , were up regulated predominantly 6 to 24 hours after stimulation.  Ref: 12887736 BMC ImmunolBMC Immunol, 2003
- Expression of IL 8, MCP 1, MCP 3, and IP 10  [ **CXCL10** ]   [ **CXCR3** ]  was significantly higher in pouchitis than normal pouch.  Ref: 12827410 Int J Colorectal Dis< MedlineTA>Int J Colorectal Dis, 2003
- CCL21, IFN gamma inducible protein 10 IP 10  [ **CXCL10** ]   [ **CXCR3** ] .  Ref: 12847218 J Immunol, 2003
- This review discusses chemokine responses in relation to HSV infection of the cornea emphasizing the role of  **CXCR3**  chemokine signaling by the IFN gamma inducible ligands MIG, IP10  [ **CXCL10** ]   [ **CXCR3** ]  and I TAC and includes discussion of their potential role in immunopathology in the nervous system.  Ref: 12815542 Curr Eye Res, 2003
- Immunohistochemical analysis indicated that mac25 AGM colocalizes at least partially with SLC and IP 10  [ **CXCL10** ]   [ **CXCR3** ]  at the basal lamina of HEVs.  Ref: 12847218 J Immunol, 2003
- In contrast, IL 4 augments gene expression and secretion of IP 10  [ **CXCL10** ]   [ **CXCR3** ]  from 1 30 1070 to 2 30 1720 pg ml 4 x 10 5 cells P <.  Ref: 12797540 J Clin Immunol, 2003
- There were significant correlations between MIG and interferon gamma IFN gamma or IP 10  [ **CXCL10** ]   [ **CXCR3** ]  levels p <.  Ref: 12856973 Acta Paediatr, 2003
- Lesional keratinocytes also overexpressed the ligands of  **CXCR3** , namely, MIG CXCL9,  **CXCL10** , and I TAC CXCL11.  Ref: 12819030 Am J Pathol, 2003
- The expression of MCP 1, MCP 3 and IP 10  [ **CXCL10** ]   [ **CXCR3** ]  were significantly lower after antibiotic treatment.  Ref: 12827410 Int J Colorectal Dis< MedlineTA>Int J Colorectal Dis, 2003
- IP 10  [ **CXCL10** ]   [ **CXCR3** ]  and MIG protein expression in mammary gland tissues was analysed by immunohistochemistry.  Ref: 12856973 Acta Paediatr, 2003
- CONCLUSION IP 10  [ **CXCL10** ]   [ **CXCR3** ]  and MIG in human milk, probably derived from milk cells and mammary gland epithelial cells, may contribute to the migration and activation of intestinal T lymphocytes to enhance mucosal immunity during the early neonatal period.  Ref: 12856973 Acta Paediatr, 2003
- Furthermore, the presence of CCL5 and  **CXCL10**  in the cytolytic granules of tissue infiltrating CD8 T cells expressing CCR5 and  **CXCR3**  reveals a potential self recruiting mechanism involving activated effector cytotoxic T cells.  Ref: 12819030 Am J Pathol, 2003
- However, 2 of 5 cases of HL like ATLL with EBV infection and 2 of 2 HL NS with EBV had IP10  [ **CXCL10** ]   [ **CXCR3** ]  MIG positive H& ; RS cells.  Ref: 12866030 Int J CancerInt J Cancer, 2003
- Upon binding with mac25 AGM, SLC and IP 10  [ **CXCL10** ]   [ **CXCR3** ]  retained all their Ca 2 signaling activity in vitro, suggesting that mac25 AGM can hold and present chemokines in the basal lamina of HEVs.  Ref: 12847218 J Immunol, 2003
- RESULTS Significant amounts of IP 10  [ **CXCL10** ]   [ **CXCR3** ]  and MIG were detected in human milk.  Ref: 12856973 Acta Paediatr, 2003
- RESULTS Expression of MCP 1, RANTES, MIP 1alpha, MIP 1beta, IP 10  [ **CXCL10** ]   [ **CXCR3** ]  and Mig was demonstrated in all Hashimoto s and most Graves thyroid specimens but very little expression was detected in the nonautoimmune goitre samples.  Ref: 12864798 Clin Endocrinol (Oxf), 2003
- In the lesions, the expression of MCP 1 mRNA was most prominently elevated, and those of RANTES and IP 10  [ **CXCL10** ]   [ **CXCR3** ]  mRNAs also increased, and their proteins were localized in tubular epithelium.  Ref: 12834626 Exp Mol Pathol, 2003
- METHODS IP 10  [ **CXCL10** ]   [ **CXCR3** ]  and MIG levels of colostrum, early milk, mature milk and sera were measured by enzyme linked immunosorbent assay ELISA.  Ref: 12856973 Acta Paediatr, 2003
- Recombinant murine interferon gamma rmIFN gamma inhibited the growth of EMT6 cells, but not K1735 cells in vitro, and strongly induced the expression of the antiangiogenic chemokine interferon inducible protein 10  [ **CXCL10** ]  IP 10  [ **CXCL10** ]   [ **CXCR3** ]  by both cell lines.  Ref: 12866028 Int J Cancer, 2003
- Tumor necrosis factor alpha TNF alpha, macrophage inflammatory protein 2 MIP 2, and interferon gamma inducible protein 10  [ **CXCL10** ]  IP 10  [ **CXCL10** ]   [ **CXCR3** ]  genes were upregulated, with two distinct peaks of mRNA expression occurring at 6 hr and 5 days.  Ref: 12804145 Hum Gene Ther, 2003
- High levels of RANTES CCL5, MCP 1 CCL2, IP 10  [ **CXCL10** ]   [ **CXCR3** ]   **CXCL10** , and Mig CXCL9 simultaneously with mononuclear cell infiltration in the lungs was found.  Ref: 12875978 Am J Pathol, 2003
- The concentrations of IP 10  [ **CXCL10** ]   [ **CXCR3** ]  and MIG in colostrum and early milk were significantly higher than those of sera from healthy controls or lactating mothers.  Ref: 12856973 Acta Paediatr, 2003
- The biopsies were taken in the first 3 months or from the 9th to the 12th month following transplantation.RESULTS IP 10  [ **CXCL10** ]   [ **CXCR3** ] , Mig, RANTES,  **CXCR3** , and CCR5 expression levels were increased in the later versus earlier biopsies P< ; =0.01 despite no change in histologic rejection grade status.CONCLUSION These results demonstrate significantly increased expression of T cell chemoattractants in heart allografts during later rejection when compared with episodes occurring shortly after transplantation.  Ref: 12829909 Transplantation, 2003
- IFN gamma inducible protein 10 IP 10  [ **CXCL10** ]   [ **CXCR3** ]  and  **CXCR3**  are highly expressed at sites of colitis.  Ref: 12874231 J Immunol, 2003
